# Involvement of autophagy in the outcome of mitotic catastrophe

**DOI:** 10.1038/s41598-017-14901-z

**Published:** 2017-11-06

**Authors:** Irina V. Sorokina, Tatiana V. Denisenko, Gabriela Imreh, Pyotr A. Tyurin-Kuzmin, Vitaliy O. Kaminskyy, Vladimir Gogvadze, Boris Zhivotovsky

**Affiliations:** 1 Faculty of Fundamental Medicine, MV Lomonosov Moscow State University, 11999 Moscow, Russia; 2 Division of Toxicology, Institute of Environmental Medicine, Karolinska Institutet, Box 210, 17177 Stockholm, Sweden; 3 Present Address: Department of Biosciences and Nutrition, Karolinska Institutet, 17177 Stockholm, Sweden

## Abstract

Evading cell death is a major driving force for tumor progression that is one of the main problems in current cancer research. Mitotic catastrophe (MC) represents attractive platform compromising tumor resistance to current therapeutic modalities. MC appeared as onco-suppressive mechanism and is defined as a stage driving the cell to an irreversible destiny, i.e. cell death via apoptosis or necrosis. Our study highlights that MC induction in colorectal carcinoma cell lines ultimately leads to the autophagy followed by apoptosis. We show that autophagy suppression in *Atg 13* knockout non-small cell lung carcinoma cells lead to the dramatic decrease of MC rate. Furthermore, mitochondria-linked anti-apoptotic proteins Mcl-1 and Bcl-xL play a crucial role in the duration of MC and a cross-talk between autophagy and apoptosis. Thus, the suppression of apoptosis by overexpression of Mcl-1 or Bcl-xL affected MC and lead to a significant induction of autophagy in HCT116 wt and HCT116 14-3-3σ^−/−^ cells. Our data demonstrate that MC induction is a critical stage, in which a cell decides how to die, while mitochondria are responsible for the maintaining the balance between MC – autophagy – apoptosis.

## Introduction

The elimination of tumor cells by commonly used anticancer drugs is mediated by triggering cell death, predominantly apoptosis, autophagy or necroptosis. However, tumors often evade cell death by affecting regulatory pathways, thereby, enhancing survival potential of tumor cells. The failure to undergo cell death in response to anticancer therapy can result in drug resistance that is a major problem limiting the effectiveness of anticancer treatment^1,2^.

Much research, therefore, has been focused on the discovery of pathways to circumvent this resistance in order to improve the treatment of cancer patients. Stimulation of mitotic catastrophe (MC) represents an important physiological process that might overcome tumor cells chemo- and radioresistance^3^. Originally, MC was described as a cell death-related process that is caused by aberrant mitosis^4^. Further, it was defined as a mechanism that senses mitotic failure resulting in an irreversible stage, such as apoptosis, necrosis or senescence. The aberrant mitosis can be caused either by premature or inappropriate entry into mitosis or a failure of mitotic checkpoints in combination with cellular damage^5,6^. MC was suggested to function as an onco-suppressive mechanism and the evasion of MC constitutes one of the gateways to cancer development^7^.

Recent studies have suggested that, similar to apoptosis, autophagy might act as an important mechanism for the regulation of cancer development and progression and in determining the response of tumor cells to anticancer therapy. Activation of autophagy occurs in response to various anticancer agents, such as temozolomide, dexamethasone, 6-thioguanine, and camptothecin, as well as to ionizing radiation^8–11^. Macroautophagy (hereafter autophagy) is an essential, conserved lysosomal degradation pathway that controls the elimination of protein aggregates and damaged organelles^12^. Autophagy can function as either a promoter or a suppressor of cancer, which makes it a promising and challenging therapeutic target. Stimulation of apoptotic and autophagic pathways often occur in the same cell and there are several molecular links between these processes^13^. The dialogue between survival and death pathways of autophagy influences the normal clearance of damaged or dying cells, as well as immune recognition of dead cell antigens. However, the role of autophagy in cancer resistance and its interplay with other types of cell death remains obscure.

The important function in cell death execution is assigned to mitochondria, which not only supply energy, control redox homeostasis and innate immunity, but are also essential for oncogenic signaling. Eliminating mtDNA limits tumorigenesis, and rare human tumors with mutant mitochondrial genomes are benign^14^. Thus, since mitochondria play a central and multifunctional role in malignant tumor progression, the targeting mitochondria might provide therapeutic opportunities^14,15^. Here we show that induction of MC in colorectal carcinoma cell lines leads to stimulation of both, autophagy and apoptosis. Moreover, the suppression of apoptosis by overexpression of Mcl-1 or Bcl-xL affected MC and led to a significant induction of autophagy in HCT116 wt and HCT116 14–3–3σ^−/−^ cells. Importantly, our data demonstrate that MC induction is a critical stage, in which a cell decides how to die, while mitochondria are responsible for the maintaining balance between MC – autophagy – apoptosis.

## Results

### Appearance and consequences of mitotic catastrophe in wild type and 14-3-3σ^−/−^ HCT116 cells

**Figure 1:**
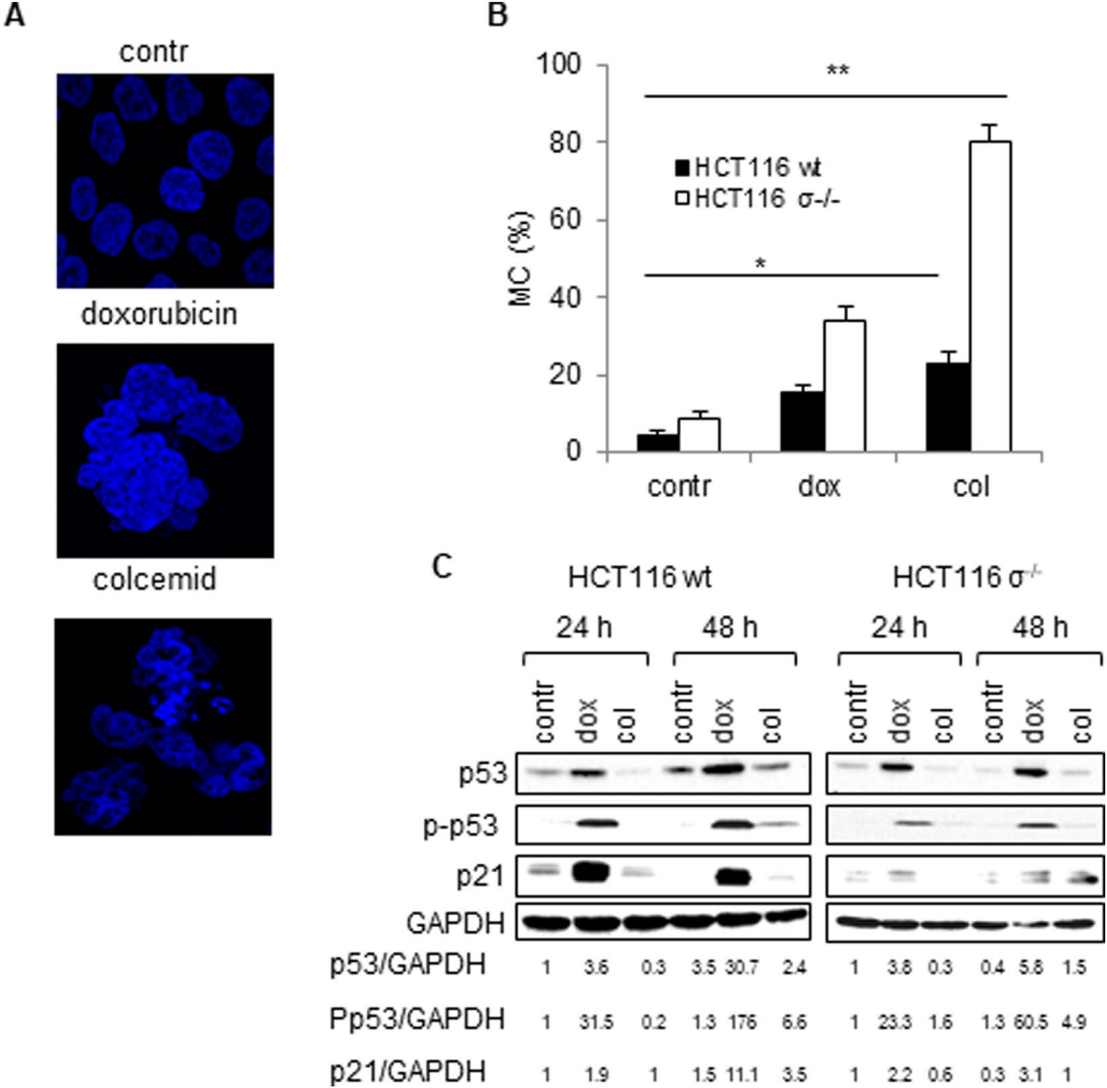
Appearance and consequences of mitotic catastrophe (MC). Wild type HCT116 and HCT116 14-3-3σ^−/−^ cells (HCT116 σ^−/−^) were treated with doxorubicin (600 nM) and colcemid (0.1 µg/ml) for 24 h. (**A**) – Representative images of MC-associated nuclear morphology. (**B**) – Quantification of MC after 24 h treatment with doxorubicin or colcemid. (**C**) – Assessment of the level of p53, P-p53, and p21 in wt HCT116 and HCT116 σ^−/−^ cells. GAPDH was used as a loading control. Densitometry analysis of p53, P-p53, and p21 bands performed and obtained data were normalized according to GAPDH using the ImageJ. Data presented as mean ± SEM, n = 12 for each cell type; *p < 0.01 (U test).

Investigation of MC in molecular terms have mostly been based on the experiments performed using HCT116 human colorectal carcinoma cells treated with doxorubicin^4,16^. In these studies, the inhibition of checkpoint kinase 2 (Chk2) or the absence of the cell cycle regulator 14-3-3σ was found to facilitate the translocation of the cyclin-dependent kinase 1 (CDK1) – cyclin-B complex to the nucleus, thereby, preventing G2 arrest and triggering MC because of entry into mitosis with unrepaired DNA. In our experiments MC in wt and 14-3-3σ-knockout HCT116 cells was induced by DNA-damaging drug doxorubicin and anti-mitotic drug colcemid. Doxorubicin is an anti-cancer drug targeting topoisomerase-II and, hence, altering DNA repair, causing cell cycle arrest at G2/M. Colcemid is also anticancer agent, which interferes with microtubule polymerization and arrests cells in metaphase. Treatment of both cell lines with sub-lethal doses of doxorubicin (600 nM) and colcemid (0.1 µg/ml) for 24 h caused accumulation of cells with specific MC-related morphology (Fig. 1A). The evaluation of MC development was assessed after Hoechst staining by counting nuclei displaying morphological changes characteristic of MC (Fig. 1B). As expected, HCT116 14-3-3σ^−/−^ cells were more sensitive to MC induction than wt cells. The amount of cells with MC morphology was markedly higher after treatment with colcemid as compared to doxorubicin administration. Thus, in response to doxorubicin, MC was detected in about 30% of HCT116 14-3-3σ^−/−^ cells, and in less than 15% of wt cells. Colcemid administration induced MC in about 80% of HCT116 14-3-3σ^−/−^ cells and only in 20% of wt cells (Fig. 1B). In order to confirm stimulation of MC, a two-dimensional assay to discriminate cells in G2 or M was performed (Fig. S1). The presence of cells in mitosis during and after treatment with drug was documented using the specific marker Ser10-phosphorylated histone H3, together with PI staining of DNA. Control (untreated) wt and 14-3-3σ^−/−^ HCT116 cells were in G2-M with no sign of polyploidy (Fig. S1), whereas treated cells showed time-dependent increase of polyploidy ( > 4 N cells). The polyploidy developed earlier and was more pronounced after colcemid administration as compared to doxorubicin (Fig. 1B, S1). At that time, the level of cyclin B upon treatment with colcemid was increased indicating MC development (Fig. S2A).

Comparison of the expression of cell cycle regulators and marker-proteins, activated upon DNA damage, revealed that doxorubicin markedly stimulated expression of p53 and its downstream target p21. Doxorubicin treatment also enhanced the level of phosphorylated p53 both in wt and HCT116 14-3-3σ^−/−^ cells. All these changes were weaker in cells treated with colcemid (Fig. 1C). Both drugs administration stimulates γH2A.X formation foci which is more explicit after colcemid treatment (Fig. S2B). It seems that doxorubicin and colcemid engage distinct pathways in MC: doxorubicin triggers the p53-dependent pathway, while colcemid is presumably acting through the mitochondria. It has been shown previously, that disintegration of microtubule system not necessarily triggers p53 activation^17,18^. Thus, it is not surprising that under conditions of our experiments, activation of p53 after treatment with colcemid was much lower than in the presence of doxorubicin.

### MC and mitochondrial activity

**Figure 2:**
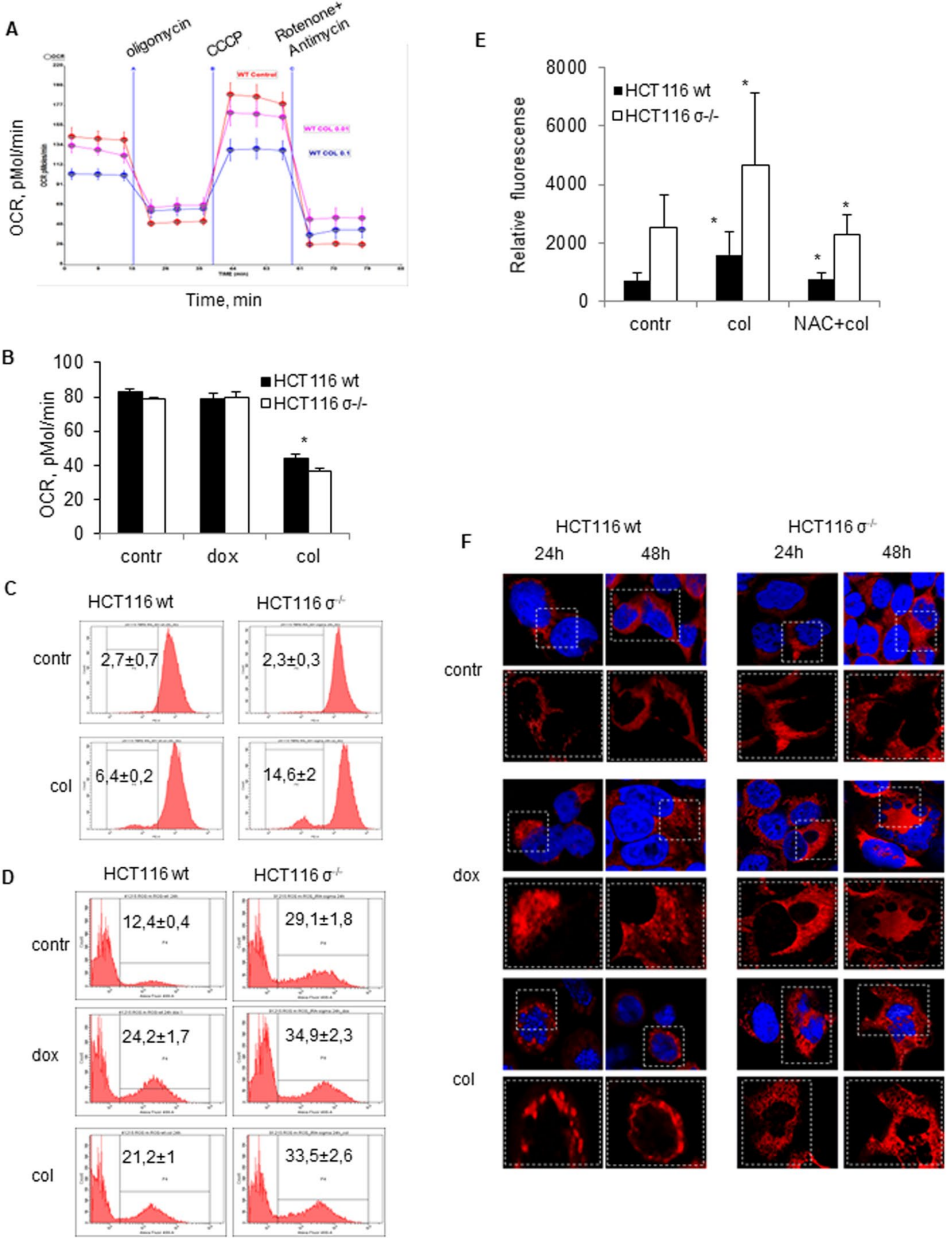
MC and mitochondrial activity. (**A**) – Representative image of oxygen consumption rate (OCR) assessment in wt HCT116 cells after treatment with colcemid (0.1 µg/ml, 0.01 µg/ml) for 24 h; (**B**) - Quantification of OCR data; (**C**) – Mitochondrial membrane potential (MMP) in wt HCT116 and HCT116 σ^−/−^ cells treated with colcemid (0.1 µg/ml) for 24 h; (**D**,**E**) – ROS level in wt HCT116 and HCT116 σ^−/−^ cells after treatment with doxorubicin (600 nM) or colcemid (0.1 µg/ml) for 24 h. In (**D**) – ROS level was measured after transfection with pHyPer-dMito plasmid, in (**E**) – using MitosoxRed; (**F**) – representative data of mitochondrial structure remodeling after treatment with doxorubicin (600 nM) and colcemid (0.1 µg/ml) for 24 h. Mitochondria were stained by MitoTracker® Red CMXRos. Magnified area outlined by white dotted rectangle. In (**B,E**) – data presented as mean ± SEM, n = 3 for each cell type; *p < 0.05 (U test).

Earlier data showed that MC is not a separate mode of cell death, rather a process (pre-stage) preceding cell death, which can occur through apoptosis or necrosis^19^. Considering the importance of mitochondria in cell death initiation and execution, the analysis of mitochondrial activity upon MC induction was performed. To this end, the assessment of mitochondrial oxygen consumption in cells treated with doxorubicin or colcemid was carried out using Seahorse Analyzer. A typical assessment of mitochondrial activity is shown in Fig. 2A. Mitochondrial respiration was relatively resistant to this concentration of doxorubicin, whereas colcemid suppressed oxygen consumption in both wt and in HCT116 14-3-3σ^−/−^ cells (Fig. 2B). A sub-population of cells with decreased membrane potential is larger in HCT116 14-3-3σ^−/−^ cells, although the difference in the extent of suppression of respiration by colcemid was non-significant (Fig. 2C). It should be mentioned that in addition to the rate of respiration other factors, such as permeability of the inner mitochondrial membrane to protons, can contribute to dissipation of the membrane potential. Unfortunately, the fluorescence spectrum of doxorubicin and TMRE (which was used for the potential analysis) are almost completely overlapping. Considering that doxorubicin did not affect mitochondrial respiration, no dramatic decrease in the membrane potential could be expected. We found elevated level of reactive oxygen species in HCT116 14-3-3σ^−/−^ cells (see below), which can cause oxidation of membrane lipids and dissipate the membrane potential. A malfunctioning respiratory chain is a source of reactive oxygen species (ROS) which can be involved in the regulation of cell death pathways. Assessment of ROS upon MC induction was carried out using pHyPer-dMito, a genetically encoded fluorescent indicator for mitochondrial H_2_O_2_ (Fig. 2D) and MitoSOX™ Red, an indicator of mitochondrial superoxide radicals (Fig. 2E). Stimulation of MC by colcemid or doxorubicin led to the mitochondrial ROS formation in both cell lines. Interestingly, a basal level of ROS in HCT116 14-3-3 σ^−/−^ cells was significantly higher than in wt cells. Oxidative stress is associated with alterations in mitochondrial structure and functions. Under the conditions employed, treatment with doxorubicin or colcemid caused remodeling of mitochondrial structure and fragmentation of elongated mitochondria (Fig. 2F). Alterations in mitochondrial structure are an important step in autophagy induction, since the formation of mitochondrial vesicles is involved in autophagosome formation^20^.

### Analysis of cell death parameters after MC induction

**Figure 3:**
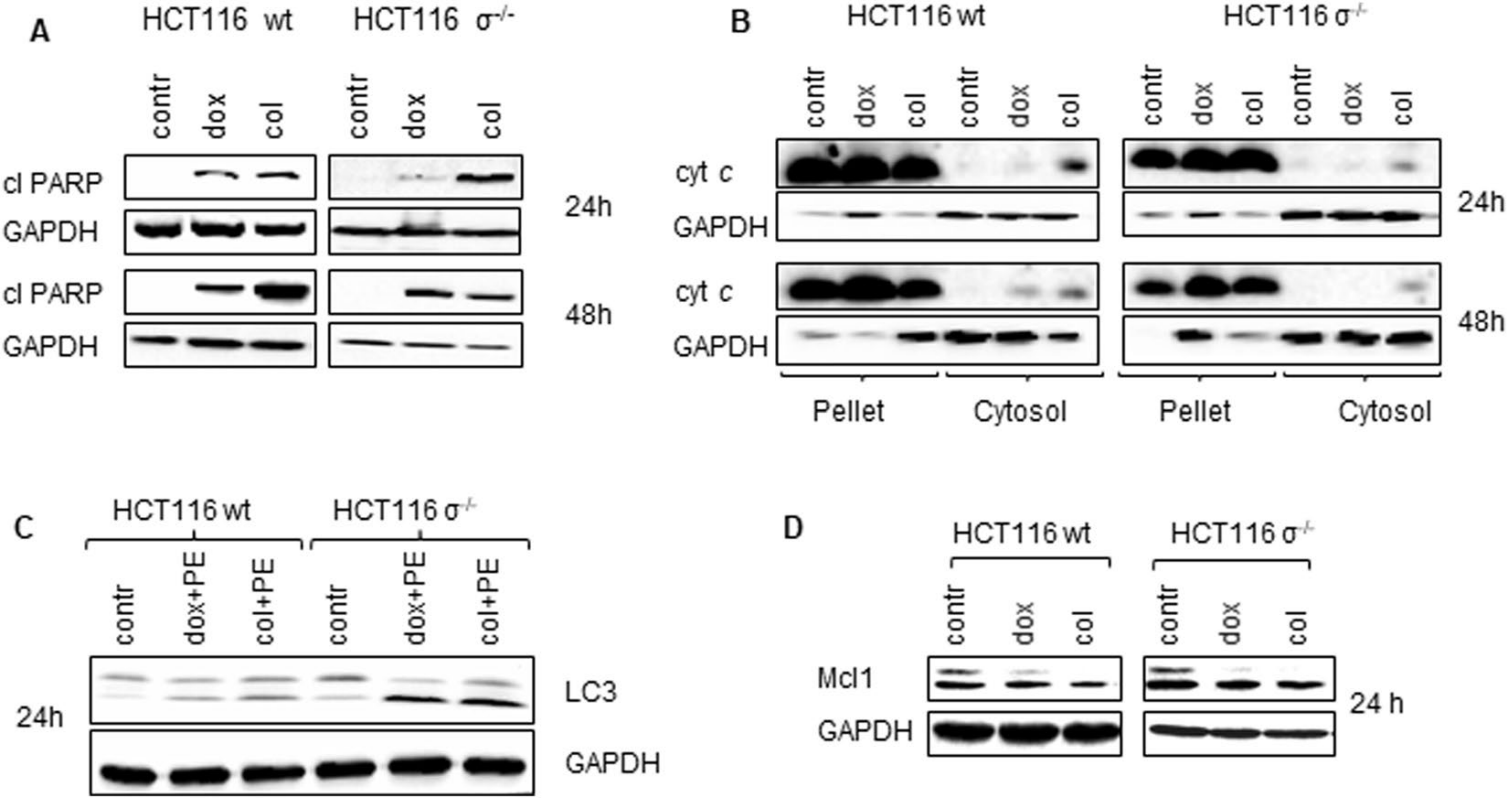
Analysis of cell death parameters after MC induction. (**A**) – wt HCT116 and HCT116 σ^−/−^ cells were treated with doxorubicin (600 nM) or colcemid (0.1 µg/ml) and cell lysates were probed by western blot for cleaved PARP, (**B**) – cytochrome *c* (cyt *c*) level in membrane and cytoplasmic fractions of HCT116 wt and HCT116 σ^−/−^ cells, treated as indicated (24, 48 hours with or without chemotherapeutic agents). (**C**) – wt HCT116 and HCT116 σ^−/−^ cells were treated with pepstatin A (5 µg/ml) + E64D (2 µg/ml), 1 h after doxorubicin (600 nM) or colcemid (0.1 µg/ml) was added. Cell lysates were probed by western blot for lipidation of LC3. PE: pepstatin + E64D. (**D**) - wt HCT116 and HCT116 σ^−/−^ cells were treated with doxorubicin (600 nM) or colcemid (0.1 µg ml) and cell lysates were analyzed by western blot for Mcl-1. GAPDH was used as a loading control.

In order to find out whether MC can prime cells to any of death modalities, the analysis of apoptotic indicator, cleavage of PARP, which is a downstream target of caspase-3, was performed after treatment with both doxorubicin and colcemid (Fig. 3A). Accumulation of cleaved PARP was more prominent in wt HCT116 cells treated with colcemid than with doxorubicin. The amount of cleaved PARP increased with time after treatment, suggesting the activation of caspase cascade in cells. Investigation of upstream mechanisms leading to caspase-3 activation revealed that MC induction stimulated low, but detectable cytochrome *c* release from intermembrane space of mitochondria (Fig. 3B). Apparently, mitochondrial outer membrane permeabilization (MOMP) occurred in a limited subset of mitochondria following MC progression (Fig. 3B). It has been shown previously that minority of mitochondria can undergo MOMP in a stress-regulated manner leading to limited caspase activation^21^. This caspase activity might promote further DNA damage through the caspase-activated DNase (CAD/DFF40). The increased level of DNA damage in MC-cells can play a role of an “amplification loop” contributing to MC development and cell death induction.

Recently, it has been shown that DNA damage can also lead to autophagy induction^22^. Indeed, assessment of LC3 processing in wt HCT116 cells and cells lacking 14-3-3σ^−/−^ protein, revealed that doxorubicin and colcemid stimulated autophagy in both cell lines, which was more prominent in HCT116 14-3-3σ^−/−^ as compared to wt cells (Fig. 3C). Apoptosis and autophagy may be linked to each other; for instance, Mcl-1, an anti-apoptotic Bcl‐2 family protein, is rapidly degraded following stress and initiates autophagy response^23^. Certainly, under the conditions employed in our experiments, the increased MC level led to Mcl-1 degradation (Fig. 3D) implying a possible pathway for autophagy activation.

Taken together, these results demonstrate that apoptosis and autophagy are consequences of MC induction. Moreover, the autophagy stimulation was more prominent in HCT116 14-3-3σ^−/−^ cells as compared to HCT116 wt implying the dependence on MC (Fig. 3C).

### Modulation of MC by Bcl-xL and Mcl-1 overexpression

**Figure 4:**
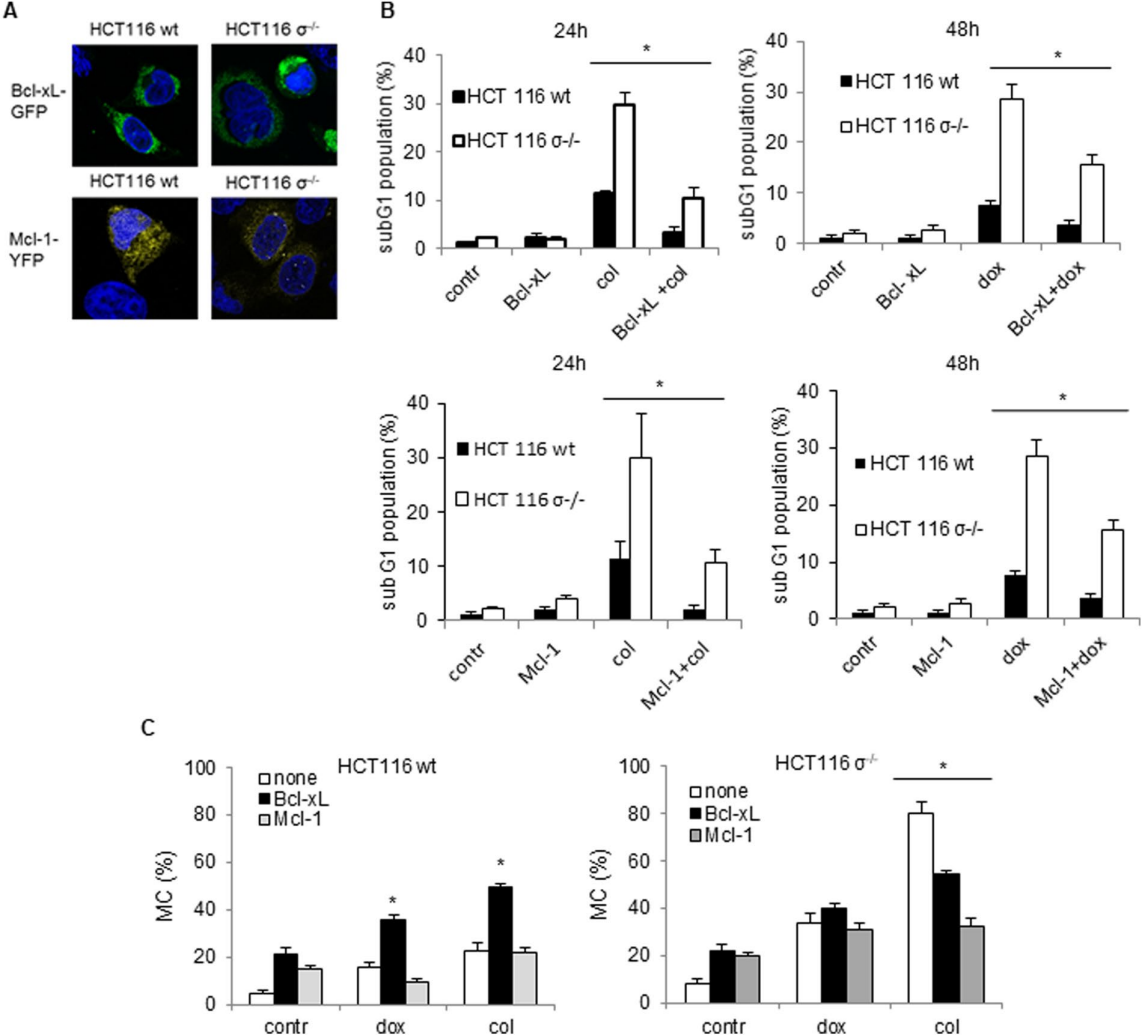
Modulation of MC by Bcl-xL and Mcl-1 overexpression. (**A**) – Representative images of cells transfected with pEYFP-C1-Dest-hMcl-1 or pEGFP-Bcl-xL-N1 plasmids. (**B**) – Apoptotic cell death, assessed by the analysis of sub-G1 population in cells treated with doxorubicin (600 nM) or colcemid l (0.1 µg/ml) 24 h after colcemid and 48 h after doxorubicin treatments. (**C**) – The development of MC in transfected wt HCT116 and HCT116 σ^−/−^cells treated with doxorubicin and colcemid for 24 h. In B, data presented as mean ± SEM, n = 5 for each cell type; *p < 0.05 (U test); in C, mean ± SEM, n = 12 for each cell type; *p < 0.01 (U test).

Since MC can lead to both apoptosis and autophagy, next we investigated whether mitochondria are involved in regulation of this balance. Both processes involve pro- and anti-survival Bcl-2 family proteins. In order to answer how these proteins can modulate the outcome of MC, wt HCT116 cells and HCT116 14-3-3σ^−/−^ cells were transfected with plasmids encoding Bcl-xL-GFP or Mcl-1-YFP (Fig. 4A), and MC was stimulated as described above. Transfection with both proteins led to suppression of doxorubicin- and colcemid-induced apoptosis assessed by the appearance of subG1 cell population (Fig. 4B). Importantly, transfection affected MC induced by doxorubicin or colcemid, but differently in wt and HCT116 14-3-3σ^−/−^ cells (Fig. 4C). Overexpression of the anti-apoptotic Bcl-xL protein stimulated doxorubicin- or colcemid-induced MC in wt cells, while transfection with Mcl-1 slightly suppressed MC development in case of doxorubicin but enhanced in case of colcemid (Fig. 4C). In HCT116 14-3-3σ^−/−^ cells, transfection with both Bcl-xL and Mcl-1 did not affect doxorubicin-, but significantly suppressed colcemid-induced MC (Fig. 4D).

### Modulation of autophagy and apoptosis by Bcl-xL and Mcl-1 overexpression

**Figure 5:**
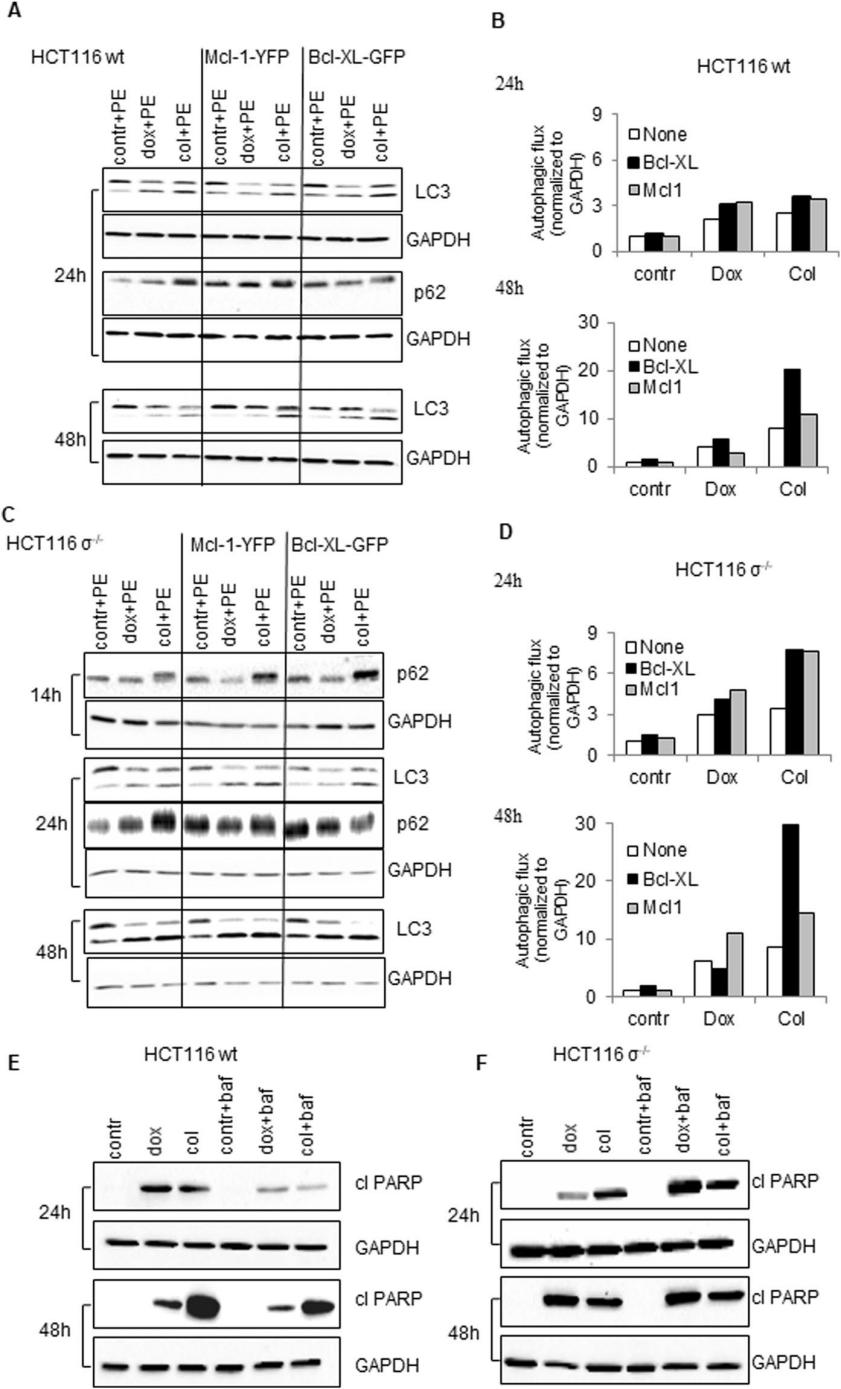
Modulation of autophagy by Bcl-xL and Mcl-1 overexpression. (**A**,**C**) – HCT116 wt and HCT116 σ^−/−^ cells were transfected with pEYFP-C1-Dest-hMcl-1 or pEGFP-Bcl-xL-N1 plasmids, after 6 h they were co-treated with pepstatin A (5 µg/ml) and E64D (2 µg/ml). After 1 h, doxorubicin (600 nM) or colcemid (0.1 µg/ml) were added. Autophagy-associated proteins were detected in cell lysates by immunoblotting at indicated time points. (**B**,**D**) – Densitometry analysis of LC3 bands performed and obtained data were normalized according to GAPDH using the ChemiDoc MP. E, F - Assessment of PARP cleavage in wt HCT116 and HCT116 σ^−/−^ cells pre-treated with 100 nM bafilomycin A (baf) for 1 h, with subsequent treatment with doxorubicin (600 nM) or colcemid (0.1 µg/ml) for 14, 24 and 48 h. GAPDH was used as a loading control.

To investigate how transfection with Mcl-1 and Bcl-xL modulates autophagy upon MC induction, we examined autophagy flux by assessment of processing of LC3-I into LC3-II and expression of primary autophagic substrate p62/SQSTM1. Notably, p62/SQSTM1 is required at early stages of autophagy, contributing to the LC3 lipidation. Transfection with Bcl-xL or Mcl-1 markedly stimulated appearance of LC3-II and p62/SQSTM1 in HCT116 14-3-3σ^−/−^ cells treated with doxorubicin or colcemid (Fig. 5C,D). In wt cells, the autophagy was developed later and was lower as compared to HCT116 14-3-3σ^−/−^ cells (Fig. 5A,B). These data suggest that under the condition of MC induction, suppression of apoptosis by overexpression of Mcl-1 or Bcl-xL can cause a significant stimulation of autophagy.

To further elucidate the interplay between autophagy and apoptosis following MC induction, we examined PARP cleavage in wt HCT116 and HCT116σ^−/−^ cells upon inhibition of autophagy by bafilomycin A1, a macrolide antibiotic, acting as an inhibitor of fusion between autophagosomes and lysosomes^24^. In wt HCT116 cells the autophagy blockage by bafilomycin A1 led to a dramatic decrease of the cleaved PARP level in doxorubicin- or colcemid-treated cells (Fig. 5E). Similarly, apoptosis inhibition by ZVAD-FMK suppressed LC3 lipidation (Fig. S2). In contrast, in HCT116 14-3-3σ^−/−^ cells apoptosis suppression led to stimulation of both MC, assessed by cyclin b1 accumulation (Fig. S2), and autophagy, whereas inhibition of autophagy enhanced the content of cleaved PARP as a result of the failure to adapt to stress conditions induced by MC (Fig. 5F).

Our results undoubtedly indicate that cellular stress induced by MC triggers autophagy, which in turn facilitates apoptosis in wt HCT116 cells, whereas, in HCT116 14-3-3σ^−/−^ cells doxorubicin- and colcemid-induced MC leads to cell death via autophagy and apoptosis, which are developing in parallel.

### Interrelation between MC and autophagy

**Figure 6:**
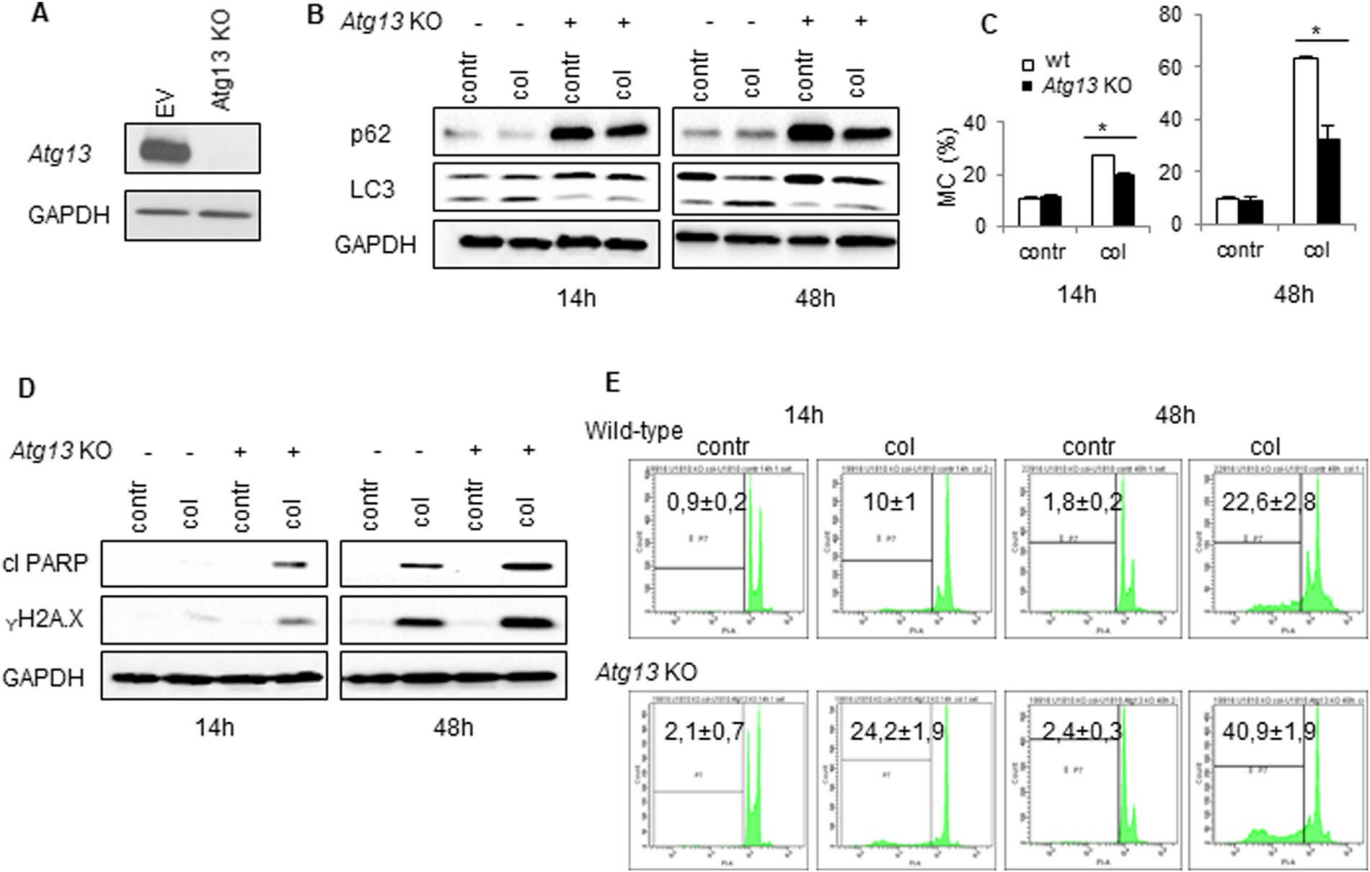
Crosstalk between MC and autophagy. (**A**) – The level of Atg13 in the generated *Atg13* KO U1810 cell line. (**B**,**D**) – Assessment of LC3, p62, γH2A.X and cleaved PARP in cells with different levels of ATG13 after treatment with colcemid (0.1 µg/ml). GAPDH was used as a loading control. (**C**) - The development of MC after 14 and 48 h treatment with colcemid in wt U1810 cells and *Atg13* KO U1810 cells. (**E**) – Apoptotic cell death assessed by the size of sub-G1 population in cells treated with colcemid (0.1 µg/ml) for 14 and 48 hours. In C, data presented as mean ± SEM, n = 12 for each cell type; *p < 0.01 (U test); in E, mean ± SEM, n = 5 for each cell type; *p < 0.01 (U test).

To confirm the interplay between MC, apoptosis and autophagy we decided to use U1810 cell line with a knockout of *ATG13* (Fig. 6A). The U1810 cell line derived from undifferentiated human non-small cell lung carcinoma was shown to be more resistant to treatment than other types of cancer. Knockout of *ATG13* efficiently blocked autophagy in these cells, as could be seen by the lack of LC3 lipidated form, LC3 II, and strong accumulation of p62/SQSTM1 (Fig. 6B). Colcemid induced MC in wt and *ATG13*-knockout U1810 cells (Fig. 6C). Thus, 48 h after treatment MC morphology was detected in more than 60% of wt U1810 cells, whereas in *ATG13*-knockout U1810 cells MC was suppressed almost two-fold (Fig. 6C). At the same time, the apoptotic response was more excessive in cells lacking *ATG13*. This was confirmed by enhanced level of PARP cleavage, amount of γH2A.X (marker of DNA degradation) (Fig. 6D) and a number of cells in sub-G1 population, which was twice as high as compared with wt cells (Fig. 6E). Thus, our data clearly indicate that there is an interrelation between MC and autophagy, which regulates efficiency of cell death execution.

After induction of MC, cells can end up either in apoptosis or in autophagy; wt HCT116 cells easier become apoptotic, whereas in HCT116 14-3-3σ^−/−^ cells autophagy prevails. Apparently, MC and autophagy can regulate each other. In U1810 cells knockout of *Atg13* indeed suppressed MC, although apoptosis was stimulated. Supposedly, the interplay between MC, autophagy and apoptosis varies from tissue to tissue.

## Discussion

Cancer cells are particularly sensitive to the induction of MC due to their common tetraploidy/aneuploidy. Cell death through MC induction may help to overcome tumor drug resistance^3^. Furthermore, elimination of tumor cells by chemotherapeutic agents requires relatively high doses; in contrast, sub-lethal dose, which is required for MC triggering, significantly limits side effects caused by cytotoxic agents^25^. Therefore, the induction of MC seems to be an attractive strategy for tumor cell elimination and the development of novel anticancer strategies. MC, as defined in 2012 by the Nomenclature Committee on Cell Death, is a *bona fide* intrinsic oncosuppressive mechanism that senses mitotic failure and responds by driving a cell to an irreversible antiproliferative fate of death: apoptosis or necrosis^26^. Mitochondria play an important role in cell death initiation and execution; however, whether mitochondria can modulate MC is unclear.

**Figure 7:**
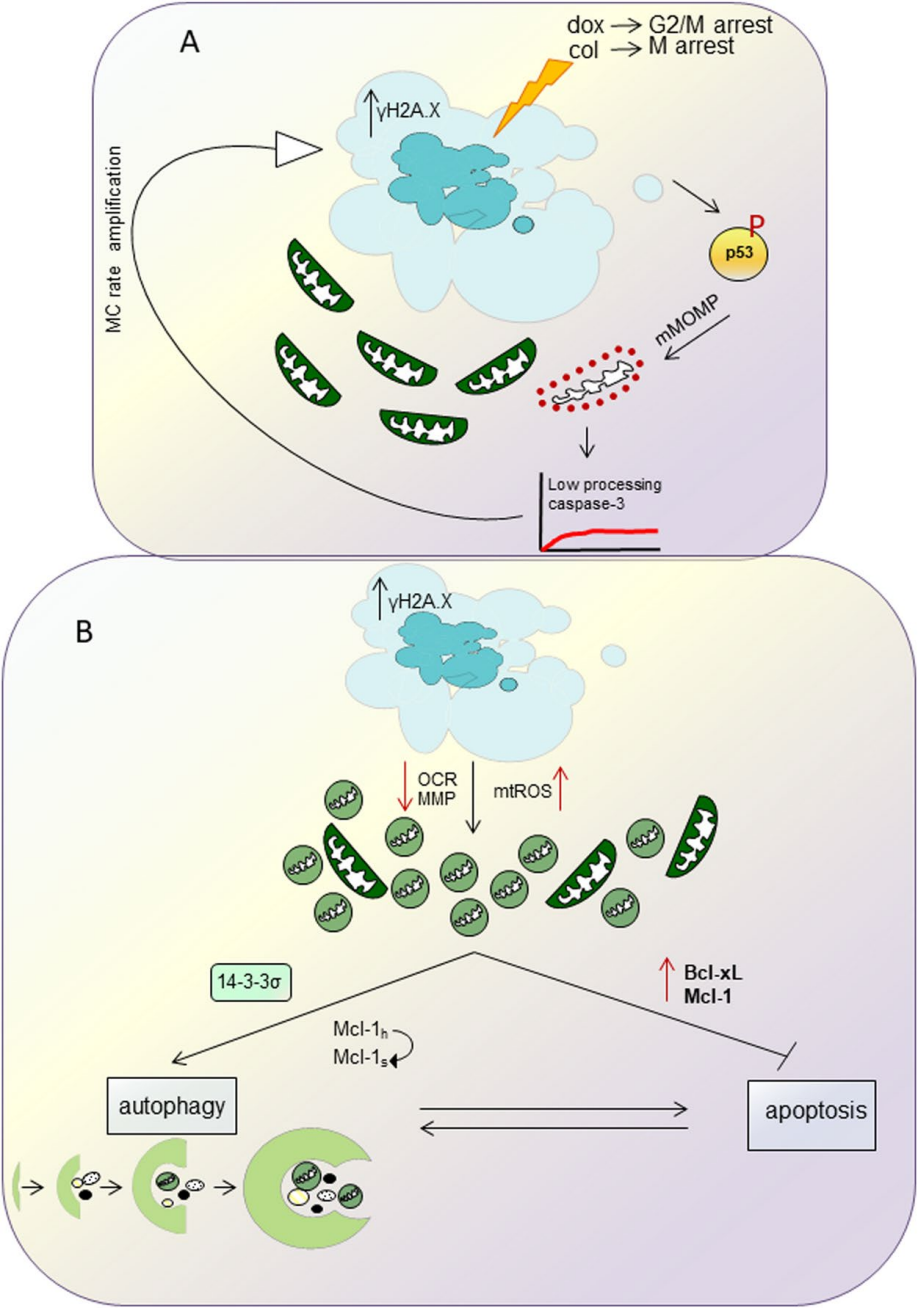
A proposed model for interplay between MC, autophagy and apoptosis orchestrated by mitochondria in cell. For the details, see the text.

Upon MC induction, expression of p53, a critical early event in the triggering cell death, was mainly observed after treatment with doxorubicin (Fig. 1 C,7). p53 controls the balance between pro- and antiapoptotic Bcl-2 family proteins and is involved in regulation of MOMP. Indeed, obtained data demonstrated that MC induction caused marginal, but detectable cytochrome *c* release (Fig. 3A). It has been shown that MOMP can occur in a small subpopulation of mitochondria (so called “minority MOMP”) leading to low level of caspase activation that promotes DNA damage^21^, and, as a result, further stimulation of MC (Fig. 7). Release of cytochrome* c* was also observed after treatment with colcemid despite a marginal stimulation of p53 expression. Apparently, mitochondrial destabilization can be explained by a direct effect of colcemid on the organelles assessed by oxygen consumption (Fig. 2A).

Colcemid-induced suppression of mitochondrial respiration was followed by a decrease in MMP (Fig. 2C) Stimulation of MC triggered ROS production, more prominent in wt cells, although the basal level of ROS in HCT116 14-3-3σ^−/−^ cells was initially higher. This can be explained by the inhibition of respiratory chain and subsequent leakage of electrons contributing to formation of ROS. In addition, induction of MC caused the alteration of mitochondrial structure and disintegration of mitochondrial network.

Apoptosis manifestations were more prominent in cells lacking 14-3-3σ^−/−^ protein. This is in agreement with other data showing that 14-3-3 protein can interact with pro-apoptotic members of Bcl-2 family proteins inhibiting their activity and supporting cell survival^27,28^. Besides, there is a correlation between 14-3-3σ and p53. Thus, 14-3-3σ was strongly induced by gamma irradiation and other DNA-damaging agents and this induction was mediated by a p53-responsive element located 1.8 kb upstream of its transcription start site. On the other hand, it has been shown that negative immunoreactivity of 14-3-3σ was significantly correlated with low p53 level. Hence, the absence of 14-3-3 protein makes cells susceptible to both MC and apoptosis.

While analyzing the consequences of MC we also found, that cellular stress triggered by doxorubicin- or colcemid caused stimulation of autophagy (Fig. 3C) (Fig. S3, S4, Movies 1–4). The enhanced autophagic flux in HCT116 14-3-3σ^−/−^cells as compared to wt HCT116 cells was correlating with MC rate (Fig. S3, S4, Movies1–4). Both, apoptosis and autophagy, are regulated by a balance between pro- and anti-survival Bcl-2 family proteins. We found that this balance also modulates the outcome of MC. Overexpression of the anti-apoptotic Bcl-xL protein stimulated MC in wt HCT116 cells in response to doxorubicin and colcemid, whereas transfection with Mcl-1 attenuated MC, especially in HCT116 14-3-3σ^−/−^ cells (Fig. 4C). In HCT116 14-3-3σ^−/−^ cells, transfection with Bcl-xL or Mcl-1 suppressed colcemid-induced MC (Fig. 4C). Different response of the Bcl-xL-transfected cells to colcemid can be explained by the role of 14-3-3σ protein in regulation of apoptotic processes. Binding 14-3-3σ to phosphorylated protein BAD releases the anti-apoptotic protein Bcl-xL from the BAD/Bcl-xL heterodimeric complex, resulting in the inhibition of apoptosis^29^. Thus, suppression of apoptosis by overexpression of Bcl-xL, leads to the accumulation of wt HCT116 cells in the state of MC. In contrast, in HCT116 14-3-3σ^−/−^ cells, overexpression of Bcl-xL and Mcl-1 suppressed MC development (Fig. 4B,C). Moreover, we found a link between MC and autophagic cell death. The cross-talk between MC and autophagy was confirmed in the experiments using U1810 cells, in which *ATG13*, an autophagy factor required for phagosome formation, was knocked out. In cells lacking *ATG13*, MC was prominently attenuated (Fig. 6). In wt cells autophagy progression was driven by dissociation of 14-3-3-hVps34 complex. 14-3-3 binding to Raptor coincides with mTORC1-ULK1-AMPK complex formation and autophagy initiation (Fig.7)^30^. In the absence of 14-3-3σ the crucial role in the balance between MC, autophagy and apoptosis play Bcl-xL and Mcl-1 proteins (Fig.7).

Autophagy stimulation after MC development represents a new approach for the improvement of anti-cancer therapy. When autophagy is unable to support cellular homeostasis and is followed by cell death, an immune response is elicited^31^. The studies using *in vivo* models have previously shown that the enhancement in the level of LC3-II promotes caspase-1 activation and IL-1β maturation^32,33^. Autophagy-competent cancers attracted dendritic cells and T lymphocytes into the tumor bed *via* release of adenosine triphosphate (ATP) from dying tumor cells^34^. Since it has been shown that autophagy has a positive impact on local immunosurveillance, this process might be important for the presentation of tumor antigens^35^. The extensive cellular stress caused by MC induction might trigger immune response in order to eliminate tumor cells (Fig.7).

In summary, MC induction leads to a new sequence of events: autophagy and subsequent apoptosis. Based on the LC3-GFP signal assessment, it seems that not all cells with MC morphology are associated with autophagy. The balanced interplay between autophagy and apoptosis is determined by MC intensity. Moreover, this interplay and MC duration are regulated by mitochondria-targeted proteins Mcl-1 and Bcl-xL. These data may represent a novel approach to the anticancer treatment, and MC may be a valid alternative in case of apoptosis impairment.

## Materials and methods

### Cell culture

The human colorectal carcinoma wt HCT116 cells (ATCC, CCL-247), 14-3-3σ-knockout HCT116 cells (gift from Prof. Bert Vogelstein, Johns Hopkins Kimmel Cancer Center, Baltimore, MD) were cultured in DMEM medium (Gibco, USA) supplemented with 10% (w/v) heat-inactivated fetal bovine serum (Gibco, USA) and 100 μg/ml penicillin and 100 μg/ml streptomycin (Gibco, USA). Human lung adenocarcinoma U1810 cells (from the collection of Uppsala University, Sweden) were cultured in RPMI 1640 (Gibco, USA) supplemented with 10% (w/v) heat-inactivated fetal bovine serum (Gibco, USA) and, 100 μg/ml penicillin and 100 μg/ml streptomycin (Gibco, USA). Cells were grown in a humidified 5% CO_2_ atmosphere at 37°C and maintained in a logarithmic growth phase for all experiments. Throughout the experiments (if not specified otherwise) cells were treated with 600 nM doxorubicin (Sigma-Aldrich, USA) or 0.1 μg/ml colcemid (Sigma-Aldrich, USA) for the indicated time periods.

### Lipofectamine-mediated transfection

For transfection pHyPer-dMito (Eurogene, Russia), pEYFP-C1-Dest-hMcl-1, pEGFP-Bcl-xL-N1 or pET28a-LC3-GFP (gift from Prof. Fazoil Ataullakhanov, Moscow) plasmids were used. Cells were transfected with the designated plasmids, using Lipofectamine LTX/Plus (Life Technologies, USA) according to the manufacturer’s instructions. Six hours after transfection the medium was changed and drugs were administrated. Cells were assayed 14, 24 and 48 hours after drug treatment.

### Construction of lenti CRISPR-*Atg13* knockout plasmid

The generation of *Atg13* knockout cell line was described in ref. ^36^.

### Hoechst staining and evaluation of mitotic catastrophe

Cells seeded overnight on coverslips were fixed for 20 minutes in 4% paraformaldehyde on ice and washed three times for 3 minutes with PBS solution. The counterstaining of nuclei was carried out by incubation for 10 minutes with Hoechst 33342 (1 μg/ml in PBS solution) at room temperature. Between all steps, cells were washed three times for 5 minutes with PBS. Stained sections were mounted using Vectashield H-1000 (Vector Laboratories) and examined under an LSM 780 confocal laser scanner microscope (Zeiss, Göttingen, Germany). Mitotic catastrophe development was evaluated by analysis of nuclear morphology under 63x/1.4 oil objective. At least 300 cells (100 transfected cells) were counted and analyzed for MC morphology for each experiment.

### Flow cytometric analysis of sub-G1 population and cell cycle analysis

For FACS analysis, cells were harvested at the indicated time points, fixed in 70% ethanol overnight and stained with propidium iodide (PI) solution [50 mg PI/ml, 0.1% (w/w) Triton X-100 and 0.1% (w/w) Na-citrate in PBS] in the presence of RNase A (0.5 mg/ml at 37°C for 30 minutes). Flow cytometric analysis was carried out using a FACS Canto II flow cytometer equipped with BD Bioscience software (Becton Dickinson, San Jose, CA).

To analyze the mitotic fraction, fixed cells were incubated with the anti-phospho-histone H3(Ser 10) antibody (Cell Signaling) followed by goat anti-rabbit IgG (H + L) secondary antibody, Alexa Fluor 488 (ThermoFisher). Stained cells were then counterstained with PI and analyzed for Alexa Fluor 488 and PI fluorescence by flow cytometry.

### Gel electrophoresis and Western-blot analysis

Cell were harvested, washed in PBS and lysed for 10 minutes at room temperature in lysis buffer supplemented with complete protease inhibitors (Roche Diagnostics). Cell extracts were centrifuged at 13,000 rpm for 20 minutes at 4°C to separate the insoluble material, followed by a determination of protein concentration using the BSA assay (Pierce). Equal amounts of protein from each sample were mixed with Laemmli’s loading buffer, boiled for 5 minutes and subjected to SDS-PAGE. Membranes were blocked for 1 hour with 5% non-fat milk in PBS at room temperature and subsequently probed with the primary antibody of interest. Blots were revealed either by ECL Western Blotting Substrate (Promega, USA) or SuperSignal West Dura Extended Duration Substrate (Thermo Scientific, USA).

### Assessment of cytochrome *c* release

Cells were digitonin-permeabilized and fractionated into supernatant and pellet. Samples were mixed with Laemmli’s loading buffer, boiled for 5 min, and subjected to 15% sodium dodecyl sulfate polyacrylamide gel electrophoresis (SDS-PAGE) at 40 mA followed by electroblotting to nitrocellulose for 30 min at 25 V. Membranes were blocked for 1 h with 5% nonfat milk in phosphate-buffered saline (PBS) at room temperature and subsequently probed overnight with a mouse anti-cytochrome *c* antibody. The membranes were rinsed and incubated with a horseradish peroxidase-conjugated secondary antibody (1:3,000). Blots were visualized by ChemiDoc MP System (Bio-rad, USA).

### Antibodies

Primary antibodies used in western blotting and immunostaining were as follows: mouse anti-cytochrome *c* (BD Biosciences, 556433); rabbit anti-phosphorylated H2A.X (Ser139) (Cell Signaling Technology, 9718); rabbit anti-human GAPDH (Trevigen, 2275-PC-100); rabbit anti-cleaved PARP (Cell Signaling Technology, 5625), rabbit anti-phospho-p53 (Cell Signaling Technology, 9284), mouse anti-TP53/p53 (Santa Cruz Biotechnology, SC-126), mouse anti-p21 Waf1/Cip1 (Cell Signaling Technology, 2946), rabbit anti-LC3 (MBL International, PM036), rabbit anti-SQSTM1/p62 (Cell Signaling Technology, 5114), anti-ATG3 (MBL International, M133-3), rabbit anti-Mcl-1 (Cell Signaling Technology, 5453), rabbit anti-phospho-histone H3 (Ser 10) (Cell Signaling Technology, 9701), rabbit anti-phospho-histone H2A.X (Ser 139) (20E3) (Cell Signaling Technology, 9718), mouse anti-cyclin B1 (Cell Signaling Technology, 4135). Horseradish peroxidase-conjugated secondary antibodies were purchased from Cell Signaling Technology.

### Assessment of oxygen consumption using Seahorse XF-96 analyzer

15,000 cells/well were grown in 96-well plates (Seahorse Bioscience, Billerica, MA), allowed to adhere overnight and treated with doxorubicin and colcemid for 24 h. Cells were then washed with assay medium (for Mitotest: unbuffered DMEM supplemented with 5 mM glucose, 2 mM glutamine, pH 7.4) before incubation with assay medium (0.175 mL) for 1 h at 37°C in a CO_2-_free incubator. Mitochondrial functional parameters were evaluated as basal respiration, proton leak after injection of oligomycin, and spare respiratory capacity after injection of the uncoupler CCCP (carbonyl cyanide 3-chlorophenylhydrazone), and non-mitochondrial respiration after injection of rotenone plus antimycin A. The data were normalized to the total protein in each well.

### Superoxide radical measurement

Cells were treated as described above and were incubated with MitoSOX™ Red mitochondrial superoxide indicator (Molecular Probes, Thermo Fisher Scientific, Carlsbad, CA, USA) according to the manufactures instruction. Then cells were immediately analyzed using a Zeiss LSM 510 META confocal laser scanner microscope (Carl Zeiss MicroImaging, Göttingen, Germany). The images obtained were quantified using ImageJ software (http://rsbweb.nih.gov/ij) and the data were calculated as corrected total cell fluorescence (CTCF), according to the equation CTCF = Integrated Density - (Area of selected cell × Mean fluorescence of background readings). The results were expressed as a fold change of the fluorescence intensity compared to the lowest value in the range of samples under comparison.

### Assessment of H_2_O_2_ production

Cells were transfected with pHyPer-dMito, after 6 h they were treated as above mentioned for indicated time periods. Cells were harvested, washed with phosphate-buffered saline (PBS). After the wash, cells dispersed in 300 μl PBS. Single-cell suspensions were kept on ice until data collection. Flow cytometry was performed on a FACS Canto II flow cytometer equipped with BD Bioscience software (Becton Dickinson, San Jose, CA). Evaluation was gated to include only living cells and cutoff thresholds for quantitation of the fluorescent cell population were determined from these experimental controls. HyPer fluorescence was acquired using 488 nm excitation laser line. Data analysis was performed using FACSDiva Software.

### Assessment of mitochondrial structure

Mitochondrial structure analysis was performed using MitoTracker® Red CMXRos (Thermo Fisher, M7512) according to manufacturer’s instructions.

### Determination of Mitochondrial Membrane Potential

Mitochondrial membrane potential (ΔΨ) was assessed using the fluorescent dye tetramethyl rhodamine ethyl ester (TMRE) in the ‘redistribution mode’. Cells were harvested, washed in PBS before being resuspended in TMRE solution (50 nM TMRE in PBS), incubated 30 min at 37°C, then, immediately analyzed on a FACS Canto II flow cytometer equipped with BD Bioscience software (Becton Dickinson, San Jose, CA) at 575 nm (FL2). A decline of mitochondria-localized intensity of fluorescence was indicative of mitochondrial membrane depolarization. Data analysis was performed using FACSDiva Software.

### Statistical analysis

The results are presented as data from three independent experiments and expressed as the mean ± S.E.M (standard error of the mean). Statistical evaluation was performed using Mann — Whitney U-test. Values of p < 0.05 were considered significant.

## Electronic supplementary material

Movie 1

Movie 3

Movie 4

Movie 2

Supplementary information-figures

Supplementary information-movies
