# Supplementary material for: Involvement of autophagy in the outcome of mitotic catastrophe
Source: Sci Rep. 2017 Nov 6;7:14571. doi: 10.1038/s41598-017-14901-z (PMC5674033; doi:10.1038/s41598-017-14901-z)
Supplement: Supplementary file 5 — Supplementary information-figures [file 41598_2017_14901_MOESM5_ESM.pdf]

Supplementary Information

Involvement of autophagy in the outcome of mitotic catastrophe

Irina V. Sorokina[1], Tatiana V. Denisenko[1], Gabriela Imreh[2,3], Pyotr A.Tyurin-Kuzmin[1], Vitaliy O Kaminskyy[2], Vladimir Gogvadze[1,2], and Boris Zhivotovsky[1,2,*]

[1]Faculty of Fundamental Medicine, MV Lomonosov Moscow State University, 11999 Moscow, Russia;

[2]Division of Toxicology, Institute of Environmental Medicine, Karolinska Institutet, Box 210, 17177 Stockholm, Sweden;

[3]Present address: Department of Biosciences and Nutrition, Karolinska Institutet, 17177 Stockholm, Sweden

*Corresponding author:
Institute of Environmental Medicine
Karolinska Institutet
Box 210
17177 Stockholm
Sweden
Boris.Zhivotovsky@ki.se

This file includes:

Supplementary Figures 1-4

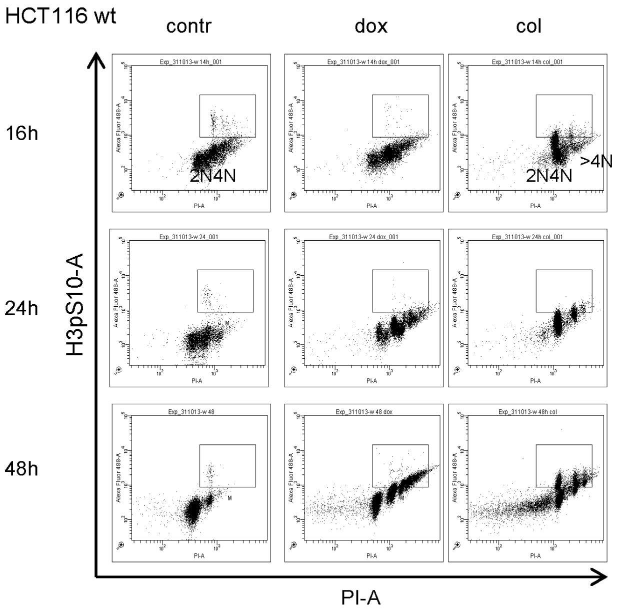

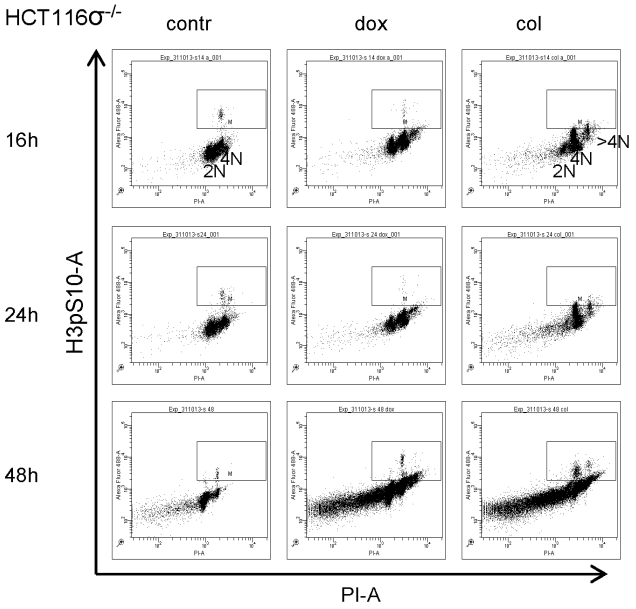

Figure S1.

Polyploidization occurs during MC. Representative images of the time-dependent polyploidy appearance (>4N cells) after treatment with doxorubicin or colcemid. Assay for the simultaneous detection of diploid and tetraploid cells by PI and cells in mitosis by an antibody against H3Sp10 (specific marker Ser-10- phosphorylated histone H3).

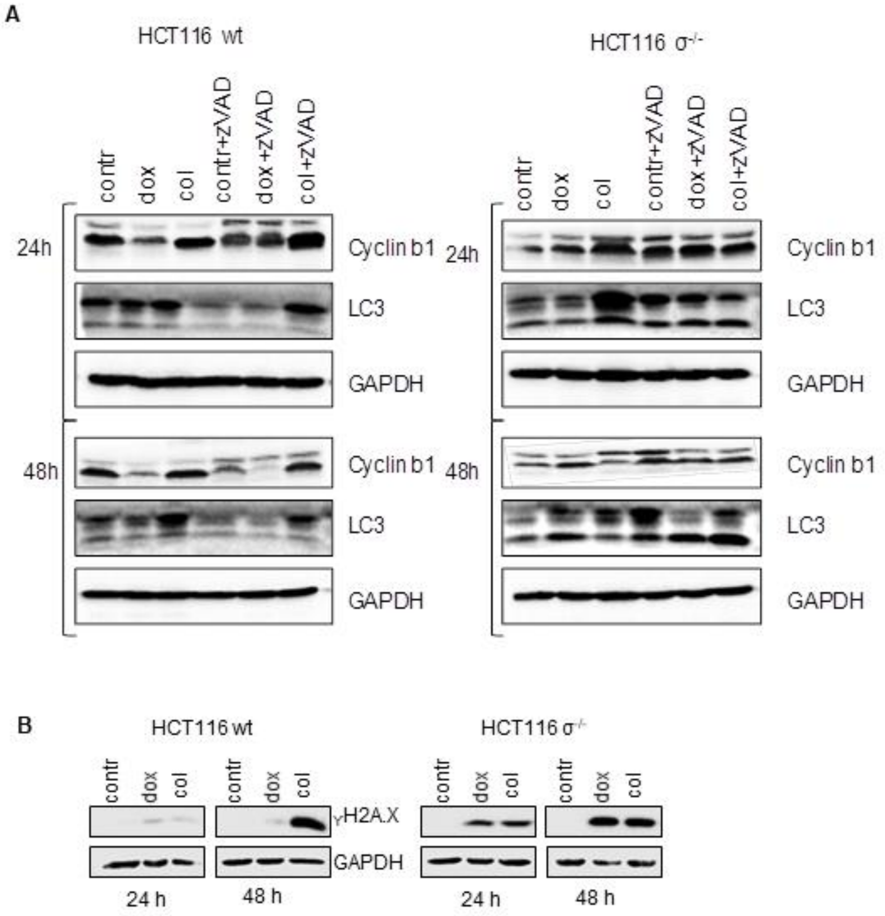

Figure S2.

Crosstalk between MC, autophagy and apoptosis. A – HCT116 and HCT116 $\sigma^{-/-}$ cells were treated with zVAD-FMK (40 µM), 1h after doxorubicin (600 nM) or colcemid (0.1 µg/ml) was added. Cell lysates were probed by western for cleavage of LC3 and level of cyclin B1. GAPDH was used as a loading control. **B -** Assessment of γH2A.X accumulation in HCT116 and HCT116 $\sigma^{-/-}$ after MC stimulation. GAPDH was used as a loading control.

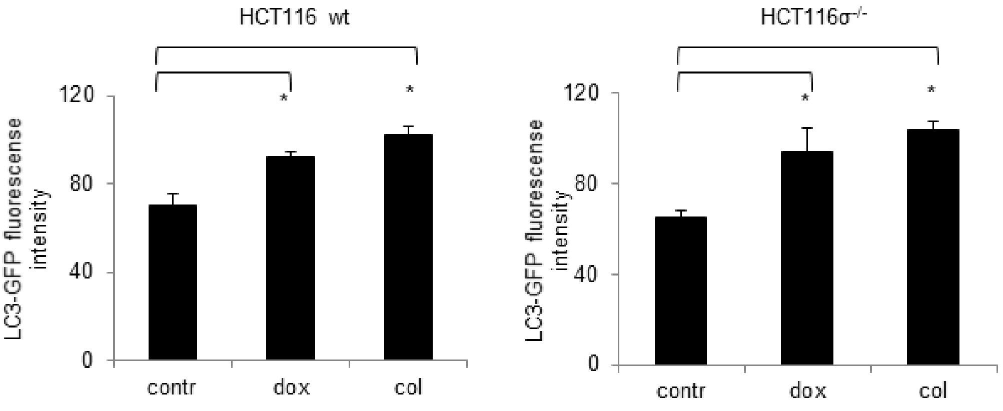

Figure S3.

MC stimulation promotes autophagy flux in HCT116 cells. HCT116 wt and HCT116 $\sigma^{-/-}$ cells were transfected with pET28a-LC3-GFP plasmid, after 6h they were co-treated with pepstatin A (5 µg/ml) and E64D (2 µg/ml). After 1h, doxorubicin (600 nM) or colcemid (0.1 µg/ml) were added. The cells were analyzed by live-imaging for 3 days at 10× magnification; scale bar, 20 µm. Each cell population calculated in the nine different fields of view. Bar chart represents the quantitation of autophagic cells with of maximum LC3-GFP fluorescence ranges. *$p<0.05$ (U test)

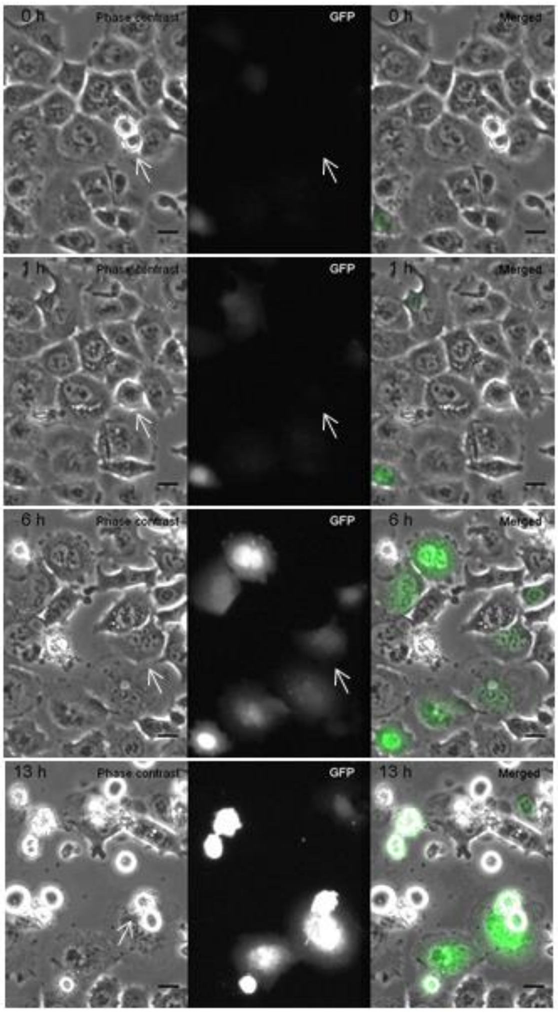

HCT116$\sigma^{-/-}$ dox

S4A

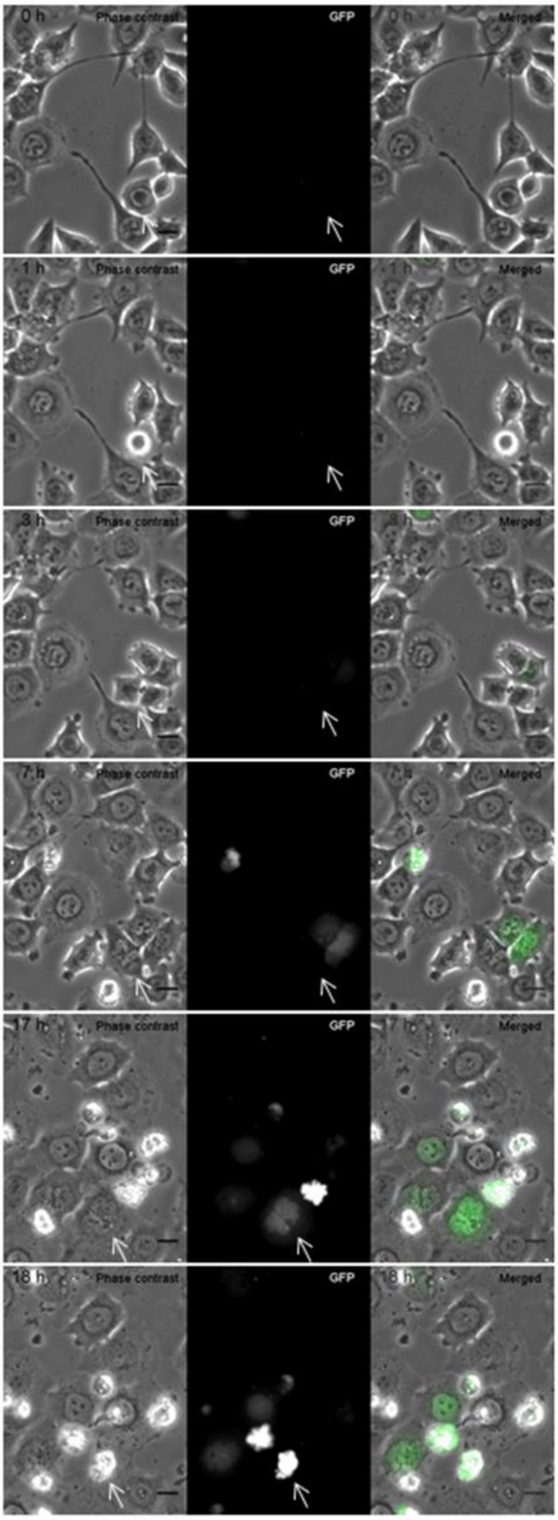

S4B

HCT116$\sigma^{-/-}$ col

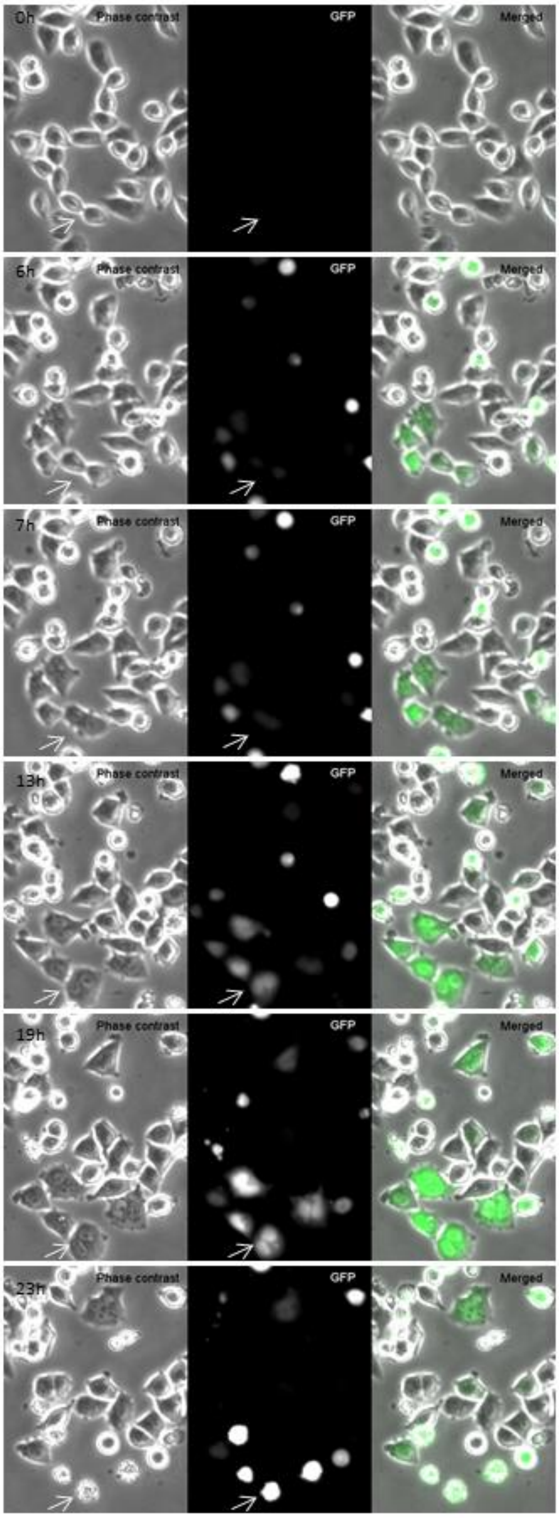

S4C HCT116 wt dox

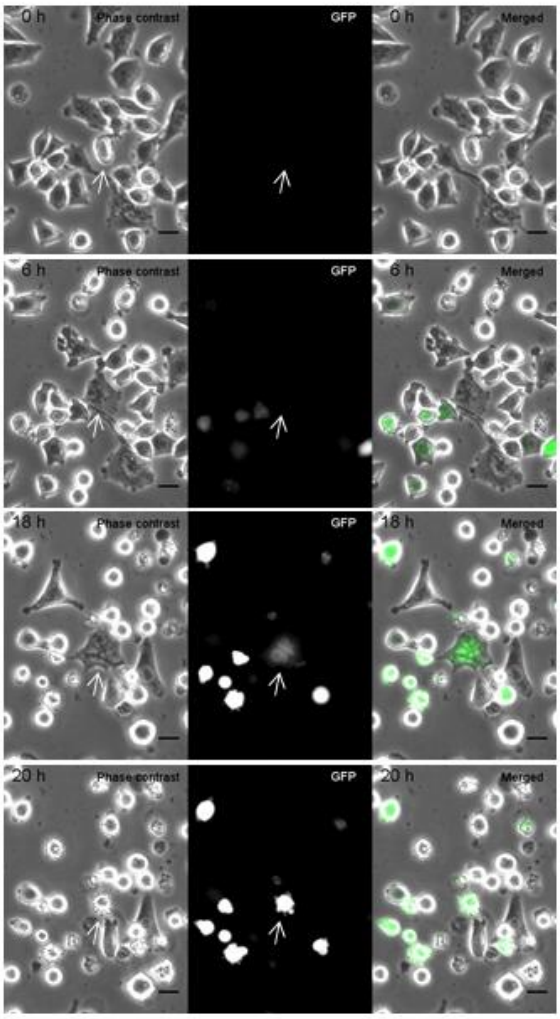

S4D HCT116 wt col

Figure S4.

Storyboard of live-imaging movies of cells undergoing MC followed by autophagy and apoptosis. HCT116 $\sigma^{-/-}$ (**A, B**) and HCT116 wt cells (**C, D**) were transfected with pET28a-LC3-GFP plasmid. After 6h they were co-treated with lysosomal inhibitors pepstatin A (5 μg/ml) and E64D (2 μg/ml). One hour later, cells were treated with doxorubicin (600 nM) (**A, C**) or colcemid (0.1 μg/ml) (**B, D**). White arrows point to cells undergoing division followed by MC. The MC stimulation promotes appearance of LC3-GFP puncta formation. MC progression and subsequent autophagy resulted in stimulation of apoptotic cell death. Live imaging of cultured cells was performed at 10× magnification; scale bar, 20 μm.
