# Supplementary material for: Involvement of autophagy in the outcome of mitotic catastrophe
Source: Sci Rep. 2017 Nov 6;7:14571. doi: 10.1038/s41598-017-14901-z (PMC5674033; doi:10.1038/s41598-017-14901-z)
Supplement: Supplementary file 6 — Supplementary information-movies [file 41598_2017_14901_MOESM6_ESM.pdf]

Supplementary Information

Involvement of autophagy in the outcome of mitotic catastrophe

Irina V. Sorokina[1], Tatiana V. Denisenko[1], Gabriela Imreh[2,3], Pyotr A.Tyurin-Kuzmin[1], Vitaliy O Kaminskyy[2], Vladimir Gogvadze[1,2], and Boris Zhivotovsky[1,2,*]

[1]Faculty of Fundamental Medicine, MV Lomonosov Moscow State University, 11999 Moscow, Russia;

[2]Division of Toxicology, Institute of Environmental Medicine, Karolinska Institutet, Box 210, 17177 Stockholm, Sweden;

[3]Present address: Department of Biosciences and Nutrition, Karolinska Institutet, 17177 Stockholm, Sweden

*Corresponding author:
Institute of Environmental Medicine
Karolinska Institutet
Box 210
17177 Stockholm
Sweden
Boris.Zhivotovsky@ki.se

This file includes:

Legends to Movies 1-4

Movie 1.

Live-imaging of HCT116 $\sigma^{-/-}$ cells treated with 600 nM doxorubicin undergoing MC followed by autophagy and apoptosis in the presence of pepstatin A (5 µg/ml) and E64D (2 µg/ml).

Movie 2.

Live-imaging of HCT116 $\sigma^{-/-}$ cells treated with 0.1 µg/ml colcemid undergoing MC followed by autophagy and apoptosis in the presence of pepstatin A (5 µg/ml) and E64D (2 µg/ml).

Movie 3.

Live-imaging of HCT116 wt cells treated with 600 nM doxorubicin undergoing MC followed by autophagy and apoptosis in the presence of pepstatin A (5 µg/ml) and E64D (2 µg/ml).

Movie 4.

Live-imaging of HCT116 wt cells treated with 0.1 µg/ml colcemid undergoing MC followed by autophagy and apoptosis in the presence of pepstatin A (5 µg/ml) and E64D (2 µg/ml).
